# The Effect of Tobacco Smoking on Bone Mass: An Overview of Pathophysiologic Mechanisms

**DOI:** 10.1155/2018/1206235

**Published:** 2018-12-02

**Authors:** Ahmad M. Al-Bashaireh, Linda G. Haddad, Michael Weaver, Xing Chengguo, Debra Lynch Kelly, Saunjoo Yoon

**Affiliations:** ^1^Ph.D., College of Nursing, University of Florida, Gainesville, FL, USA; ^2^Professor, Associate Director for Graduate Program, College of Health and Human Services, University of North Carolina Wilmington, Wilmington, NC, USA; ^3^Professor, Associate Dean for Research and Scholarship, College of Nursing, University of Florida, Gainesville, FL, USA; ^4^Professor, College of Pharmacy, University of Florida, Gainesville, FL, USA; ^5^Assistant Professor, College of Nursing, University of Florida, Gainesville, FL, USA; ^6^Associate Professor, College of Nursing, University of Florida, Gainesville, FL, USA

## Abstract

Recent evidence demonstrates that tobacco smoking causes an imbalance in bone turnover, leading to lower bone mass and making bone vulnerable to osteoporosis and fracture. Tobacco smoke influences bone mass indirectly through alteration of body weight, parathyroid hormone-vitamin D axis, adrenal hormones, sex hormones, and increased oxidative stress on bony tissues. Also, tobacco smoke influences bone mass through a direct effect on osteogenesis and angiogenesis of bone. A RANKL-RANK-OPG pathway is an essential regulatory pathway for bone metabolism and its importance lies in its interaction with most of the pathophysiologic mechanisms by which smoking influences bone mass. Both first- and secondhand smoke adversely affect bone mass; smoking cessation seems to reverse the effect of smoking and improve bone health. Recent advances in research on bone turnover markers could advance scientific knowledge regarding the mechanisms by which smoking may influence bone mass.

## 1. Introduction

Tobacco smoke has more than 7,000 chemicals, and evidence clearly demonstrates tobacco smoking causes premature death, cancer, and a variety of chronic diseases, such as coronary heart disease and chronic obstructive pulmonary disease [1, 2]. Several studies support the effects of tobacco smoking on the skeletal system. Smoking was identified as a risk factor for osteoporosis and fractures and was included in the Fracture Risk Assessment Tool. In the United States, it is projected three million fractures occur annually due to osteoporosis, with an estimated economic cost of $25.3 billion by 2025 [3].

The current research in this field shows smoking may have detrimental effects on the skeletal system. Specifically, recent evidence demonstrates tobacco smoking causes an imbalance in the mechanisms of bone turnover, leading to lower bone mass and bone mineral density (BMD) making bone vulnerable to osteoporosis [4–8] and fracture [4, 5, 7–11]. Due to the high quality of available evidence, the recent Surgeon General report causally linked tobacco smoking with several skeletal system disorders (e.g., hip fracture, rheumatoid arthritis, and periodontitis) [12].

The best way to reduce the adverse effects and cost of smoking on human health is to quit tobacco use; consequently, many cessation programs have been developed. However, these programs have only limited efficacy [13]. The reasons individuals return to tobacco use are likely multifactorial, and the emergence of new modes of smoking, such as water pipes and e-cigarettes, adds to the complexity [14]. While programs are developed to promote cessation, there is a need to address individuals who currently suffer from smoking-related bone complications. To reduce the effect of smoking on bone, more studies are required to understand the pathophysiologic mechanisms of smoking on bone health [4, 5, 8].

The major aims of this review are to (1) summarize pathophysiologic mechanisms responsible for the effect of tobacco smoke on bone mass; (2) discuss the interaction between pathophysiologic mechanisms, with respect to Receptor Activator of Nuclear Factor-Kappa B Ligand-Receptor Activator of Nuclear Factor-Kappa B-Osteoprotegerin (RANKL-RANK-OPG) pathway and bone turnover markers; and (3) understand effects of secondhand smoke and smoking cessation on bone mass.

## 2. Effect of Smoking on Bone Mass: Pathophysiologic Mechanisms

**Figure 1:**
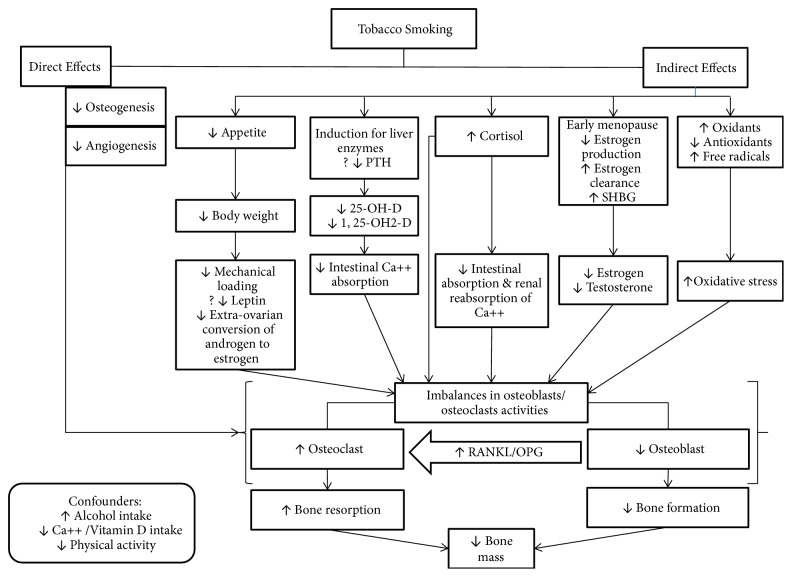
Potential pathophysiologic mechanisms of decreased bone mass in tobacco smokers. PTH: parathyroid hormone; 25-OH-D: 25-hydroxy vitamin D; 1, 25-OH2-D: 1, 25 dihydroxyvitamin D; RANKL: Receptor Activator of Nuclear Factor-Kappa B Ligand; OPG: Osteoprotegerin.

The pathophysiological mechanisms of smoking on bone health remain unclear because there are few appropriately designed studies to clarify mechanisms, and some findings are contradictory [4]. Over the last 10 years, four outstanding reviews covering mechanisms of the effects of tobacco smoke on bone health have been published [4, 5, 15, 16]. Similar mechanisms, with slight variation, were discussed in these reviews. In general, these mechanisms are classified as either* direct* or* indirect*.  Figure 1 and Table 1 summarize these pathophysiological mechanisms.  Table 2 provides summary for effects of smoking on bone health including, but not limited to BMD, bone formation and resorption markers, and the confounders for smoking effects.

### 2.1. Indirect Mechanisms

#### 2.1.1. Alteration in Body Weight

Tobacco smokers usually have lower body weight and body mass index (BMI) compared to nonsmokers, which may be explained by the suppressive effect of nicotine on appetite [17]. This relationship is not well understood because the effect of smoking on BMD and risk for fracture persists after controlling for low body weight and low BMI [4].

Wong, Christie, and Wark (2007) offered an explanation for these effects [5]; they suggested low BMI or body weight (1) decreases the effect of mechanical loading necessary to enhance osteogenesis; (2) is associated with less fatty tissue, thus the extraovarian conversion of androgen to estrogen is reduced in smokers; or (3) may be associated with lower leptin levels, that are correlated with bone mass; however, the findings here are inconsistent between smoking and serum leptin levels.

#### 2.1.2. Alteration in Parathyroid Hormone- (PTH-) Vitamin D Axis

The PTH-vitamin D axis has a key role in bone mass density and the hemostasis of calcium. PTH is a hormone controlling the serum level of ionized calcium via bone resorption and renal absorption, while the active form of vitamin D [1, 25 Dihydroxyvitamin D (1, 25-OH2-D)] regulates intestinal absorption of calcium [18, 19]. Researchers have found tobacco smoking reduces bone mass through its effect on vitamin D and calcium absorption [4]. Low 25-hydroxy vitamin D (25-OH-D) and 1, 25-OH2-D in smokers were reported in several studies [20–27], which may be related to the decreased intake of vitamin D or the induction of the liver enzyme that enhances hepatic metabolism of vitamin D metabolites [5], or via suppression of parathyroid hormone (PTH) release [16].

The effect of smoking on suppression of PTH is inconsistently reported [20–23, 25, 28]; such inconsistencies are partially rationalized as a result of the confounding effects of weight, alcohol intake, estrogen use, and the variability in intake of calcium and vitamin D [21, 29]. Also, researchers have found smoking impairs intestinal calcium absorption via changes in calciotropic hormone metabolism, remaining significantly lower in smokers despite adjustment for confounders (e.g., vitamin D and calcium supplementation, age, and sex) [30–32].

#### 2.1.3. Alteration of Adrenal Hormones

Smoking has been shown to increase cortisol level, leading to hypercortisolism [33] in chronic smokers [34, 35], though this finding has not been reported in all studies [36]. Compared to nonsmokers, smokers had higher levels of androstenedione and dehydroepiandrosterone [37, 38]. A high level of glucocorticoid in smokers alters bone metabolism and decreases bone mass either directly by changing the osteoblast and osteoclast activities or indirectly by altering the gastrointestinal absorption and renal reabsorption of calcium [39–42].

#### 2.1.4. Alteration of Gonadal (Sex) Hormones

Both estrogen and testosterone play a protective role in bone metabolism. Estrogen acts via suppression of bone resorption [43–45]; testosterone has a direct effect on bone through the androgen receptors present in osteoblasts that enhance bone proliferation [46–50], or it has an indirect effect through the aromatization changes to estrogen [51]. In women, tobacco smoking enhances estrogen metabolism resulting in a lower level of estradiol [52, 53]. Women who smoke usually experience menopause two years earlier than women who do not smoke [54, 55]. There are three proposed ways by which smoking may modify the production and metabolism of estrogen. First, nicotine, cotinine, and anabasine inhibit the aromatase enzyme, also called* estrogen synthase*, in a reversible manner and suppress the production of estrogen [56]. Second, smoking boosts the hepatic breakdown of estradiol via 2*α*-hydroxylation leading to irreversible inactive metabolite (estrone to 2-methoxyestrone) [53, 57, 58]. Third, smoking increases the level of the serum sex hormone binding globulin (SHBG) that may reduce the level of free estradiol in the blood [58, 59].

In men, there are contradictory findings; some studies found levels of testosterone were similar in both smokers and nonsmokers, while other studies found levels of testosterone were higher in smokers [35, 60–62]. Similar to women, the mechanism of aromatase inhibition was reported in men; this mechanism suppressed production of estradiol from testosterone [16].

#### 2.1.5. Increased Oxidative Stress

Tobacco smoking is associated with high levels of free radicals [63, 64] that may increase bone resorption and contribute to lower bone mass [65]. Smokers have significantly lower antioxidant enzyme levels (superoxide dismutase, glutathione peroxidase, and paraoxonase) and higher levels of oxidative stress products (malondialdehyde, nitric oxide) than nonsmokers [66]. According to a 5-year prospective large-scale study investigating the intake of two antioxidant vitamins (C and E) on smokers and nonsmokers, the risk of hip fracture in smokers with low intake of both vitamins was increased fivefold compared to nonsmokers [67].

### 2.2. Direct Effect on Bone Tissue

Smoking has a direct effect on bone tissue. Bone is dynamic tissue undergoing continuous remodeling via bone formation and resorption [16, 68]. Osteoblasts and osteoclasts are the major cells responsible for bone remodeling. The activities of both are regulated by several factors, including the RANKL-RANK-OPG pathway, estradiol, various cytokines, and calciotropic hormones [16, 45, 69]. However, Leibbrandt and Penninger (2008) reported only RANK(L) was absolutely vital for in vivo osteoclast differentiation, as evidenced by a complete absence of osteoclast in RANKL and RANK knockout mice [69].

Several receptors are involved in osteoblast and osteoclast activities, such as nicotinic acetylcholine receptors and androgen receptors in osteoblasts, and aryl hydrocarbon receptors in osteoblasts and osteoclasts [4]. The most abundant compound in tobacco is nicotine that binds to nicotinic receptors in osteoblasts. At low levels, this binding increases cell proliferation, while at higher levels it inhibits osteoblast production, resulting in cell death [70]. Nicotine has an inhibitory effect on osteogenesis and on angiogenesis that play key roles in bone metabolism [16]. An in vivo study in rabbits found nicotine had a dose-dependent inhibitory effect on osteoblast development and on vascular endothelial growth factor, necessary for angiogenesis [71]. In addition, chemical polycyclic aryl hydrocarbon compounds such as benzo(a)pyrene can bind to aryl hydrocarbon receptors in osteoblasts and osteoclasts; such constitutive binding with aryl hydrocarbon receptors may have deleterious effects on bone [4]. See Table 2 for further details.

#### 2.2.1. RANKL-RANK-OPG Pathway

RANKL and OPG are members of the superfamily of tumor necrosis factor (TNF) and TNF receptor, respectively, and their binding to receptor activator of NF-kB (RANK) has a fundamental role regulating osteoclast formation, proliferation, activity, and survival [72]. RANKL is a membrane protein mainly produced by osteoblasts. Once RANKL binds to its natural receptor, expressed by osteoclast precursor cells, it stimulates osteoclast precursor differentiation to active mature osteoclasts and accelerates bone resorption [73]. OPG, a soluble receptor also produced by osteoblasts that acts as a decoy receptor and neutralizes RANKL, prevents RANKL from interacting with RANK and consequently inhibits osteoclast proliferation, activity, and survival [73, 74].

Few studies have explored the relationship between the RNKL-RANK-OPG pathway and smoking. Laboratory studies found rats exposed to smoke inhalation had higher levels of RANKL/OPG ratio compared to control rats not exposed to smoke [16, 90]. Several studies in humans investigating the relationship between smoking, RANKL-RANK-OPG pathway, and periodontitis found smokers had a lower level of OPG [75, 76] and a higher RANKL/OPG ratio than nonsmokers [75–77]. Also, a recent human study comparing smokers and nonsmokers found smokers had significantly lower levels of OPG. Smokers have lower, but not statistically significant, levels of RANKL and higher, but not statistically significant, levels of RANKL/OPG ratio [25]. According to the authors the findings for OPG and RANKL/OPG were expected; however, the finding of lower levels for RANKL in smokers was unexpected and suggests RANKL can be affected by factors other than smoking (e.g., sex, age, gingival disease, rheumatoid arthritis, multiple myeloma, and diabetes) [25].

#### 2.2.2. Importance of the RANKL-RANK-OPG Pathway

The RANKL-RANK-OPG pathway has great influence on osteoclast formation and activities [45, 91]. The importance of the RANKL-RANK-OPG pathway is that a majority of indirect pathophysiological mechanisms (alteration in PTH-vitamin D axis, alteration of adrenal hormones (cortisol), and alteration of estrogen and testosterone) interact with this pathway affecting bone turnover and bone mass [45, 69]. This pathway interacts with other factors influencing bone turnover, such as prostaglandin E2 and interleukins [45, 69]. An example of these interactions is gonadal hormones with the RANKL-RANK-OPG pathway. There is crosstalk between the RANKL-RANK-OPG pathway and androgen, estrogen, and androgen receptors. Estrogen and androgen affect RANKL-RANK signaling and suppress the effect of osteoclast differentiation by controlling the expression of OPG and the downregulation for the cascade of JNK-c-Jun [92].

Interestingly, a knockout study in mice found that factors that stimulate or suppress bone resorption through osteoclast lineage also influence OPG and RANKL expression at the mRNA and protein levels [69]. In addition, it was confirmed that only RANK(L) was absolutely vital for in vivo osteoclast differentiation [69].

#### 2.2.3. Bone Turnover Markers

Several studies have investigated the relationship between smoking and bone mass as measured by BMD, but the trend of research in this field is to use bone turnover markers to provide insight into the dynamics of bone turnover in metabolic bone disorders, monitor effectiveness of antiresorptive therapies, and predict the earlier risk of osteoporosis and fracture [93]. Indeed, the purpose is to intervene earlier, rather than later, once changes are evident and confirmed by diagnostic imaging techniques, such as dual-energy X-ray absorptiometry [93]. Also, recent evidence demonstrates bone turnover markers can be a complementary tool to BMD, because the increment in bone turnover markers is associated with microarchitecture changes affecting bone quality and may increase fracture risk independent of BMD [68].

After bone reaches peak mass, it undergoes constant remodeling via resorption followed by formation [16, 68]. Several biomolecules are released into systemic circulation during bone resorption and formation. Those biomolecules are bone turnover markers. According to Lian and Stein (2006), under ideal physiological conditions, bone resorption occurs in 10 days, while bone formation requires around 3 months [94].

There is a broad range of bone turnover markers that reflect bone formation or resorption. Recently, the serum procollagen type I N-terminal propeptide (PINP) has been recommended as a standard marker for bone formation, while the carboxyl-terminal telopeptide of collagen type I (CTXI) has been recommended as a standard marker for bone resorption [68, 93, 95]. Both markers are recommended by the International Osteoporosis Foundation and International Federation of Clinical Chemistry [93].

Laboratory studies have found smoke-exposed male rats had significantly higher levels of TRACP and lower levels of OC and b-ALP activities than unexposed control rats [96]. In terms of human studies, few researchers have explored the relationship between smoking and bone turnover markers. One cross-sectional study found statistically significant differences in serum levels of OPG and CTXI between smokers and nonsmokers [25]. Further studies are needed to understand the effect of smoking on bone turnover markers. See Table 2 for further details.

### 2.3. Confounding Factors

Cusano (2015) recommended assessing the effects of smoking on BMD by adjusting confounding lifestyle factors [4]. Physical activity, alcohol consumption, and dietary calcium intake are examples of confounding factors [4, 5]. A number of studies have reported that smokers tend to consume more alcohol, consume less dietary calcium, and perform less physical activity than nonsmokers. These factors could contribute to lower bone density [9, 29]. While not addressing smoking, smoking effect was not considered besides other lifestyle factors, a recent review of lifestyle and osteoporosis concluded that adequate calcium/vitamin D intake, excessive exercise, and regular weight-bearing exercise are essential for bone health and reduce the risk of osteoporosis and fracture [97]. A study of elder Chinese women found a former habit of exercise was associated with a lower risk of osteoporotic fracture [78]. Another study found greater levels of weight-bearing physical activity increase total hip and femoral neck BMD (*p* ≤ 0.0001) and cortical (*p* < 0.0001) and periosteal bone volumes (*p* = 0.016) [79]. Alcohol, however, had an adverse effect on bone health. Consuming ≥ 4 glasses/day of alcohol increased the risk for fracture [98].

Overall, the findings here are inconclusive. One study reported no significant difference in calcaneus BMD between three groups: only alcohol drinkers, both alcohol drinkers and smokers, and control (nondrinker/nonsmoker). However, blood total alkaline phosphatase activity was significantly lower in the combined drinker and smoker group than a control group (*p* < 0.05). The authors reported an inverse relationship between duration of alcohol consumption and ALP levels (*p* < 0.001) and N-mid osteocalcin (*p* < 0.001) [80]. Two other studies reported similar findings regarding a lack of effect for alcohol use on BMD [81, 82]. Contrary to those findings, the protective effect of alcohol was reported in a study that found moderate alcohol consumption associated with greater BMD (*p* ≤ 0.015) [79]. Yet, in two other studies, researchers reported a negative effect of alcohol use on BMD. The first of these studies found that a former habit of alcohol consumption in women was significantly associated with a greater risk of osteoporotic fracture (OR = 2.47, 95%CI: 1.07- 5.53) [78]; the second study found lower mean BMD at age 17 in girls who had smoked and reported drinking at age 13 [83]. See Table 2 for further details.

## 3. Effect of Secondhand Smoke on Bone Health

There is evidence supporting the adverse effect of secondhand smoke on bone health. Laboratory studies in rats, mouse models, and cell culture demonstrate direct negative effects of passive smoke on osteoblast and osteoclast activities [99, 100]. Two cross-sectional studies reported that subjects exposed to secondhand smoke had significantly lower phalangeal BMD (*p* < 0.01) [84] and higher risk for femoral neck osteoporosis than unexposed subjects (OR, 3.68; 95%CI: 1.23-10.92) [85].

## 4. Effect of Smoking Cessation on Bone Health

Few studies have investigated the effect of smoking cessation on bone health. One study found an intermediate risk of fracture in ex-smokers [86]. Another study found the effect of smoking on bone density was reversible, and the bone density of ex-smokers improved in less than 10 years [87]. Interestingly, other studies reported that the effects of smoking cessation in postmenopausal women produced improvement in gonadal hormones, level of bone formation, and resorption markers in 6 weeks and improvement in the bone density after 1 year of cessation/reduction [88, 89].

## 5. Conclusion

Smoking tobacco has been associated with reduced bone mass and increased risk of fracture through its direct or indirect effects on osteoblast and osteoclast activities. The RANKL-RANK-OPG pathway plays a vital role in the mechanisms by which smoking may result in poor bone health, because this pathway has been found to mediate most pathophysiological mechanisms. This review indicates that the effect of tobacco smoke on bone health is complex and involves several mechanisms. Further research is needed to understand the various mechanisms by which smoking adversely affects bone health. Advances in the field of bone turnover will provide a means for researchers to shed light on these mechanisms.

## Figures and Tables

**Table 1:** Summary for potential pathophysiologic mechanisms of decreased bone mass in tobacco smokers.

**Mechanisms**	**References**
**Indirect mechanisms**	
**Alteration in body weight**	
Nicotine suppresses appetite.	[17]
Low BMI or low body weight (1) decreases the effect of mechanical loading necessary to enhance osteogenesis; (2) is associated with less fatty tissue, thus the extraovarian conversion of androgen to estrogen is reduced in smokers; or (3) may be associated with lower leptin.	[5]
The effect of smoking on BMD and risk for fracture persists after controlling for low body weight and low BMI.	[4]
**Alteration in parathyroid hormone- (PTH-) vitamin D axis **	
Tobacco smoking reduces bone mass through its effect on vitamin D and calcium absorption.	[4]
Low 25-OH-D and 1, 25-OH2-D in smokers were reported in several studies.	[20–27]
Low vitamin D in smokers may due to (1) the induction of the liver enzyme that enhances hepatic metabolism of vitamin D metabolites or (2) suppression of PTH release.	[5, 16]
Suppression of PTH in smokers was not consistently reported.	[20–23, 25, 28]
Smoking impairs intestinal calcium absorption via changes in calciotropic hormone metabolism, remaining significantly lower in smokers despite adjustment for confounders.	[30–32]
**Alteration of Adrenal Hormones**	
Smoking increases cortisol level.	[33–35]
Smokers had higher levels of androstenedione and dehydroepiandrosterone.	[37, 38]
A high level of glucocorticoid in smokers alters bone metabolism and decreases bone mass either directly by changing the osteoblast and osteoclast activities or indirectly by altering the gastrointestinal absorption and renal reabsorption of calcium.	[39–42]
**Alteration of Gonadal (Sex) Hormones**	
Tobacco smoking enhances estrogen metabolism resulting in a lower level of estradiol.	[52, 53]
Regarding testosterone, some studies found levels of testosterone were similar in both smokers and nonsmokers, while other studies found levels of testosterone were higher in smokers.	[35, 60–62]
Women who smoke usually experience menopause two years earlier than women who do not smoke.	[54, 55]
Smoking may modify the production and metabolism of estrogen through (1) inhibition of aromatase enzyme (*estrogen synthase*) and suppression of the production of estrogen; (2) increase in the hepatic breakdown of estradiol to irreversible inactive metabolite; and (3) increase in the level of the serum SHBG that may reduce the level of free estradiol.	[53, 56–59]
**Increased Oxidative Stress**	
Tobacco smoking is associated with high levels of free radicals.	[63, 64]
High levels of free radicals may increase bone resorption and contribute to lower bone mass.	[65]
Smokers have significantly lower antioxidant enzyme levels and higher levels of oxidative stress products than nonsmokers.	[66]
**Direct Effect on Bone Tissue**	
Polycyclic aryl hydrocarbon compounds have deleterious effects on bone.	[4]
Nicotine has an inhibitory effect on osteogenesis and on angiogenesis that play key roles in bone metabolism.	[16]
Nicotine at low levels increases cell proliferation, while at higher levels it inhibits osteoblast production, resulting in cell death.	[70]
Nicotine had a dose-dependent inhibitory effect on osteoblast development and on vascular endothelial growth factor, necessary for angiogenesis.	[71]

**Table 2:** Effect of tobacco smoking on bone health.

Citation	Design	Sample Characteristics	Study Purpose	Findings
Ward & Klesges, 2001 [6]	Meta-analysis	(i) N = 40,753 subjects from 1 86 cross-sectional studies (ii) The average of age was 53.3 years (iii) The majority were females (74%) (iv) The average of age (Mean ± SD): 18.9 ± 0.6	(i) To determine the magnitude and the association between cigarette smoking and bone mass.	(i) Compared with nonsmokers (never and former smokers), smokers had significantly reduced bone mass at all bone sites, averaging a one-tenth standard deviation deficit for combined sites. (ii) Smoking increases the lifetime risk of developing a vertebral fracture by 13% in women and 32% in men.

Law & Hackshaw, 1997 [7]	Meta-analysis	(i) N= 11,861: 2,156 smokers and 9,705 nonsmokers from 29 cross sectional studies (ii) N = 3,889 subjects from 19 cohort and case control studies that were recording hip fractures (iii) The average of age was 54.0 years (iv) The majority were females	(i) To determine the magnitude and the association between smoking, bone mineral density (BMD), and risk of hip fracture according to age.	(i) In premenopausal women bone density was similar in smokers and nonsmokers. (ii) In postmenopausal women bone loss was greater in current smokers than nonsmokers, and the bone density diminishing by about an additional 2% for every 10 year increase in age. (iii) Compared to nonsmokers, current smokers had similar risk of hip fracture at age of 50; however, such risk was increased thereafter by an estimate of 17% at age of 60, 41% at age of 70, 71% at age 0f 80, and 108% at age of 90 years.

Szulc et al, 2002 [22]	Cross-sectional study [sample from MINOS study]	(i) N = 719: 231 never smokers, 405 former smokers, and 83 current smokers (ii) The range of age was 51-85 years (iii) All participants were males (iv) Random sample	(i) To determine the effect of smoking on BMD and bone turnover.	(i) Current and former smokers had similar BMD, except for the forearm. (ii) Compared with never smokers, former smokers had lower BMD at most skeletal sites. (iii) The three groups of subjects did not differ in terms of levels of bone formation markers; however, levels of bone resorption markers (urinary C-terminal telopeptide and free and total deoxypyridinoline) were higher in current smokers than in former and never smokers. (iv) Compared with former and never smokers, current smokers had lower level of 25-hydroxyvitamin D.

Lorentzon et al, 2007 [24]	Cross-sectional study [sample from GOOD study]	(i) N = 1,068: 975 nonsmokers, 93 smokers (ii) The average of age (Mean ± SD): 18.9 ± 0.6 for nonsmokers and 19.0 ± 0.6 years for smokers (iii) All participants were young males	(i) To investigate if smoking habit associated with bone size and areal or volumetric BMD (aBMD or vBMD).	(i) Smokers had significantly lower aBMD of the total body, lumbar spine, and trochanter than nonsmokers. (ii) Smokers had lower cortical thickness of both the radius and tibia than the nonsmokers, whereas no difference was seen for cortical vBMD. (iii) Smokers had higher levels of testosterone (total and free) and lower 25-OH-D than nonsmokers and the adjustment for such differences did not alter the associations between smoking and bone parameters.

Kargin et al, 2016 [25]	Cross-sectional study	(i) N = 170: 85 nonsmokers and 85 smokers (ii) The average of age ( Mean ± SD): 43.58 ± 6.58 nonsmokers and 43.52 ± 6.72 smokers (iii) All participants were males	(i) To compare of the bone turnover markers between smoker and nonsmoker male.	(i) Smoker's C-terminal telopeptide (CTX) level was significantly lower than that of the nonsmokers (0.30 ± 26.97 ng/ml vs. 65.10 ± 42.41 ng/ml, *p* = 0.007) (ii) Smoker's mean serum Parathyroid Hormone (PTH) level was significantly lower than that of nonsmokers (23.75 ± 9.88 pg/ml vs. 31.35 ± 13.15 pg/ml, *p* ≤ 0.001), and parallel findings were observed for vitamin D (16.75 ± 8.73 ng/ml vs. 19.50 ± 8.97 ng/ml, *p* = 0.044).

Kassi et al, 2015 [26]	Cross-sectional study	(i) N = 181: 117 nonsmokers and 64 smokers (ii) The average of age ( Mean ± SD): 34.69 ± 7.38 with a range of age 20-50 years (iii) All participants were males (iv) Random sample	(i) To determine the prevalence of vitamin D (25-OH-D, D-2 and D-3) insufficiency and its association with smoking, BMD, and bone markers.	(i) The prevalence of 25-OH-D < 20ng/ml) was 50.3%. (ii) There was a strong correlation between 25-OH-D and smoking (*P* < 0.001); 25-OH-D level was significantly lower in smokers than nonsmokers. (iii) In total population, regardless of the age group, there was no correlation between 25-OH-D and BMD in the femoral neck and the lumbar spine. Also, 25-OH-D was not correlated with bone turnover markers: serum osteocalcin, P1NP, b-CTXs levels.

Christie et al, 2009 [27]	Cross-sectional study	(i) N = 69 twin (ii) Average of age (Mean ± SD): 53 ± 8.9 with a range of (40-76) years (iii) 13 were males, and 56 were females	(i) To examine whether mechanism of bone loss in pair twins could be related to smoking.	(i) Percentage within-pair difference (WPD) that was calculated based on the differences between smokers and nonsmokers were found to be significant for BMD of femoral neck (-5.6%, 95%CI: -9.0 to -2.2, *p* = 0.002), total hip (- 6.2%, 95%CI: -9.4 to -2.9, *p* ≤ 0.001), and whole body bone mineral content (BMC) (- 4.1%, 95%CI: -7.2 to -1.1, *p* = 0.012). However, it was found to be not significant for lumbar spine (-3.5%, 95%CI:-7.0 to 0.0, *p* = 0.058), and forearm (-0.8%, 95%CI: -2.6 to -1.0, *p* = 0.290). (ii) WPD for fat mass was also lower in smoking twins (-12.8%, 95%CI:-20.7 to -4.8, *p* = 0.005), and lean mass marginally significant (-2.8, 95%CI: -5.9 to 0.3, *p* = 0.083).

Fujiyoshi et al, 2016 [28]	Cross-sectional study	(i) N = 376: 240 never smokers, 64 former smokers,72 current smokers (ii) The range of age was 24-36 years (iii) 205 were males, and 171 were females (iv) 181 were white, 195 were black	(i) To examine whether smoking was associated with serum parathyroid hormone (PTH) independent of correlates of PTH among young adults and explore potential mechanisms.	(i) Compared to nonsmokers, current smoker had lower PTH and there was no evidence of an interaction by race and sex. (ii) The lowest level of PTH was detected in current smokers followed by former smokers (intermediate level), while the highest was seen in never smokers (mean of PTH: 23.6, 26.7, 27.4 pg/mL, respectively: *p* for trend = 0.006, adjusted for calcium intake confounders). (iii) Current smoker had the lowest biomarkers concentration of serum osteocalcin and 24-hour urinary excretion of calcium.

Krall & Dawson-Hughes, 1999 [31]	Randomized-placebo-controlled study, subjects followed for 3 years	(i) N = 402: 370 nonsmokers, 32 smokers (ii) The average of age (Mean ± SD): 71 ± 5. nonsmokers, and 70 ± 2 smokers (iii) 45% were males, and 55% were females	(i) To determine the relationship of smoking to rates of BMD change and to intestinal calcium absorption.	(i) Compared with nonsmokers, smokers had significant higher adjusted annualized rates of BMD loss at the femoral neck, and total body; meanwhile, no significant difference was observed at the spine. (ii) The adjusted mean of calcium absorption fraction was lower in smokers than nonsmokers.

Rapuri et al, 2000 [32]	Cross-sectional study	(i) N = 444: 390 nonsmokers, 54 smokers (21: heavy smokers, 33: light smokers) (ii) The range of age was 56-77 years (iii) All participants were females	(i) To examine the relationship between smoking and BMD, calciotropic hormones, calcium absorption.	(i) The adjusted mean total body bone mineral density was 4% and the total hip density was 6% lower in heavy smokers than that of nonsmokers. (ii) Compared with nonsmokers, light and heavy smokers had lower level for the adjusted mean calcium absorption. (iii) Compared with nonsmokers, heavy smokers had significantly lower level of serum 25-OH-D. (iv) Compared with nonsmokers, heavy smokers had significantly higher levels of serum osteocalcin and urine N-telopeptide/creatinine ratio.

Cetin et al, 2009 [66]	Cross-sectional study	(i) N = 60: 30 nonsmokers, and 30 smokers (ii) The average of age (Mean ± SD): 51.2 ± 3.4 for nonsmokers, 49.7 ± 3.5 for smokers (iii) All participants were postmenopausal women (iv) Participants were randomly selected	(i) To investigate the impact of smoking on the oxidative status in postmenopausal women, and to assess the relationship between BMD and oxidant/antioxidant parameters.	(i) The rates of osteopenia and osteoporosis in smokers and nonsmokers were 75% and 52.5%, respectively. (ii) The T-scores were significantly lower in smokers than nonsmokers (median: -2.7 vs. -1.4, *p* < 0.001). (iii) Activities of antioxidant enzymes (superoxide dismutase, glutathione peroxidase, paraoxonase) were lower and the levels of oxidative stress products (malondialdehyde, nitric oxide) were higher in smokers than in nonsmokers (*p* < 0.001). (iv) In the smoking group, there was a significant correlation between decreased T-score and oxidative stress parameters.

Melhus et al, 1999 [67]	Prospective case-control study	(i) N = 66,651 [44 case developed fracture and 93 age-matched of current smokers] (ii) The range of age was 40-76 years (iii) All participants were females	(i) To determine whether the dietary intake of antioxidant vitamins may modify the increased risk for hip fracture among the smoker.	(i) The odds ratio (OR) for hip fracture among current smokers with a low intake of vitamin E was 3.0 (95% CI: 1.6-5.4), and of vitamin C 3.0 (95%CI: 1.6-5.6), and it was increased to 4.9 (95%CI: 2.2-11.0) with a low intake of both vitamins E and C. (ii) The OR decreased to 1.1 (95%CI: 0.5-2.4) and 1.4 (95%CI: 0.7-3.0) with high intakes of vitamin E and C, respectively. Such effect was not observed for beta-carotene, selenium, calcium, or vitamin B6.

Tang et al, 2009 [75]	Cross-sectional study	(i) N = 149 periodontitis patient: 58 never, 39 former, and 52 current smokers (ii) The range of age was 26-86 years (iii) 56 were males, and 93 were females	(i) To compare the levels of the sRANKL, OPG and their relative ratio in gingival crevicular fluid (GCF) among periodontitis patients with varying smoking histories.	(i) There were no significant differences for sRANKL, OPG, and their relative ratio among never smokers, former smokers, and current smokers. (ii) Compared to never smokers, high pack-years group had significantly reduced OPG and subsequently increased sRANKL/OPG ratio (positively correlated with pack-year even after adjustment for age and status of current smoking).

Lappin et al, 2007 [76]	Cross-sectional study	(i) N = 70: 35 nonsmokers, and 35 smokers (ii) The average of age (Mean, Range): 43.0 (40.0 -51.5) nonsmokers, 43.0 (41.0 - 50.5) for smokers (iii) 46 were males, and 24 were females	(i) To compare serum levels RANKL and OPG in age- and sex-matched groups of smokers and nonsmokers with identical levels of periodontal disease.	(i) Compared to nonsmokers, smokers had significantly lower median serum level of OPG (23.76 pM vs. 59.28 pM, *p* = 0.0006) but not for RANKL (41.47pM vs. 48.23 pM, *p* = 0.0942). (ii) Tobacco consumption had statistical significant negative correlation with the concentrations of OPG.

Ozcaka et al, 2010 [77]	Cross-sectional study	(i) N = 86: 44 with CP (31 nonsmokers, 13 smokers), 42 healthy control group (29 nonsmokers, 13 smokers) (ii) The range of age was 35-65 years for group of CP, and 33-57 for healthy control group (iii) 43 were males (23 CP, 20 control), and 43 were females (21 CP, 22 control)	(i) To evaluate plasma levels sRANKL and OPG in smoker versus nonsmoker CP patients.	(i) All periodontal measurements were significantly different between the two groups of healthy control and group of CP (*p* < 0.05). However, these measurements were not differed between smoker and nonsmokers of CP group (*p* > 0.05). (ii) Chronic periodontitis smokers exhibited significantly lower plasma OPG concentrations (*P* = 0.007) and higher sRANKL/OPG ratio (*p* = 0.01) than healthy control smokers.

Du et al, 2011[78]	Cross-sectional study	(i) N = 703: 281 nonsmokers, 422 smokers (former and current) (ii) Mean of age was 93.48 years (iii) 226 were males, and 477 were females	(i) To observe the relationships of osteoporotic fracture with habits of smoking, tea consumption, alcohol consumption, and exercise among very old unrelated Chinese nonagenarians and centenarians.	(i) In older Chinese people, there were significant associations between the increased risk of osteoporotic fracture and current or former alcohol drinking, and the risk for osteoporotic fracture was significantly reduced with habit of former exercise. Smoking and tea consumption were found not to be associated with osteoporotic fracture. (ii) The former habit of alcohol consumption was significantly associated with a greater risk of osteoporotic fracture (OR = 2.47, 95%CI: 1.07- 5.53), but the former habit of exercise was associated with a lower risk of osteoporotic fracture.

Eleftheriou et al, 2013 [79]	Retrospective cohort study	(i) N = 723 healthy male military recruits: 329 nonsmokers, 41 Ex-smokers, 35 recent Ex-smokers, and 244 current smokers (ii) The average of age was 19.92 with a range of 16-18 years. (iii) All participants were Caucasian males	(i) To investigate the influence of young men lifestyles factors of smoking, alcohol, and physical activity on the peak bone mass as evidenced by the changes on the bone structure and geometry.	(i) Smoking was associated with well-maintained bone geometry, but worse BMD (*p* = 0.0001) and calcaneal quantitative ultrasound (QUS) (*p* ≤ 0.0005). (ii) Alcohol consumption at moderate level was associated with higher BMD (*p* ≤ 0.015). (iii) The increment in weight-bearing exercise was associated with improved periostial bone apposition, total hip, and femoral neck BMD (*p* ≤ 0.0001) at cortical (*p* < 0.0001) and periostial level (*p* = 0.016).

Kim et al, 2007 [80]	Cross-sectional study	(i) N = 463: alcohol-only drinking (n = 254), combined alcohol drinking and smoking (n = 125), and control nondrinking/nonsmoking (n = 84) (ii) Average of age was 23 and the range of age was 20-26 years (iii) All participants were Korean males	(i) To investigate effects of alcohol and tobacco smoking on BMD and bone metabolism.	(i) There were no significant differences in BMD of the calcaneus among the 3 groups. However, blood total alkaline phosphatase activity (ALP) was significantly lower in the combined drinking and smoking group than in the control group (*p* < 0.05). (ii) There were negative relationships between duration of alcohol consumption and ALP N-mid osteocalcin levels (all *p* < 0.001). (iii) Daily cigarette use and smoking duration showed a significantly negative correlation with ALP (*p* < 0.001).

Dorn et al, 2011 [81]	Cross sequential design	(i) N = 262: 171 nonsmokers, 91 smokers (ii) The average of age (Mean ± SD): 14.9 ± 2.2 years (iii) All participants were females (iv) The majority were Caucasian (61.8%) or African American (32.8%) with some mixed race/other (5.4%)	(i) To examine the association between depressive and anxiety symptoms, smoking, and alcohol use on bone health whether the association between depressive and anxiety symptoms varied by smoking or alcohol use individually or by combined use.	(i) The higher state of depressive symptoms was associated with lower BMC and BMD. (ii) Participants with lowest use of smoking had higher BMD (hip, femoral neck), however; no differences were observed by alcohol use. (iii) Compared with alcohol (regular, nonusers), the regular users of both cigarettes and alcohol had a stronger negative association between depressive or anxiety symptoms and total body BMC.

Dorn et al, 2013[82]	Cross sequential design	(i) N = 262: 171 nonsmokers, 91 smokers (ii) The average of age (Mean ± SD) at time 1: 14.35 ± 2.16 years (iii) All participants were females (iv) 32% black, and 62% white	(i) To examine the impact of depressive and anxiety symptoms, smoking, and alcohol use on bone accrual in girls 11-19 year with age cohort of 11, 13, 15, and 17 years.	(i) The lower rate of lumbar spine and total hip BMD of ages 13-19 were associated with higher frequency of smoking (ii) There was an association between high depressive symptoms and lower lumbar spine BMD across 11-19 years of age. (iii) Alcohol intake and anxiety had no effect on bone outcome, and depressive symptoms had no effect on total body BMC.

Lucas et al, 2012 [83]	Prospective cohort study	(i) N = 731: at age 13 years, one fourth tried smoking, while 59% used alcohol and 20% had both (ii) The range of age was 13-17 (iii) All participants were females	(i) To quantify the short-and long-term associations between smoking and alcohol drinking initiation and bone mineral density in adolescent girls.	(i) Lower mean BMD was observed at age of 17 years (late adolescence) in girls who had ever smoked by 13, and similar trend was observed for those consumed alcohol at that age.

Holmberg et al, 2011 [84]	Cross-sectional study	(i) N = 15, 038 underwent bone mineral density (BMD) scan: 5, 829 exposed to home passive smoking in their adulthoods (ii) Mean age of 52.7±13.8 [range: 18-95] (iii) 40% were males, and 60% were females	(i) To investigate the association between phalangeal BMD and self-reported passive smoking.	(i) Subjects who have been exposed to passive smoking at home as an adult had significantly lower BMD than unexposed subjects (0.343 vs. 0.331 g/cm2; *p* < 0.01), even when adjusted for age, gender, weight, height, smoking (pack-years; 0.339 vs.0.337 g/cm2; *p* < 0.05)

Kim et al, 2013 [85]	Cross-sectional study	(i) N = 925 never smokes: 212 with secondhand smoke (SHS), and 713 without SHS (ii) Mean age of 51.4 ± 9.9 years (iii) Mean of age (mean ± SD): 64.6 ± 7.1 for subject with SHS, and 66.3 ± 7.8 for subject without SHS	(i) To assess the association between SHS and postmenopausal osteoporosis.	(i) Compared to participants not exposed to SHS, participants who actively exposed to SHS from family members had higher adjusted OR for femoral neck osteoporosis (OR: 3.68; 95%CI: 1.23-10.92). (ii) Compared with the non-exposed group, the group who lived with cohabitant smokers had increased risk for lumbar and femoral osteoporosis regardless of the number of cigarettes consumed by their cohabitant.

Cornuz et al, 1999 [86]	Prospective cohort study	(i) N = 116,229 [377 case of fracture] (ii) The range of age was 34-59 years (iii) All participants were females	(i) To examine effects of cigarette smoking and smoking cessation on the risk of hip fracture in women.	(i) Compared with never smokers, age-adjusted relative risk (RR) of hip fracture among current smokers was 1.3 (95%CI: 1.0 to 1.7). (ii) There was a linear association between the risk of hip fracture and cigarette consumption (*p* = 0.09). (iii) After 10 years, former smokers had a reduced risk of hip fracture (adjusted RR = 0.7, 95%CI: 0.5 to 0.9) compared with current smokers.

Gerdhem & Obrant, 2002[87]	Cross-sectional study [sample from OPRA study]	(i) N = 1,042 [377 case of fracture] (ii) All participants age was 75 years (iii) All participants were females (iv) Participants were randomly selected	(i) To asses effects of cigarette-smoking on bone mass.	(i) Compared to never smokers, current smokers had lower BMD for femoral neck (0.71 vs. 0.76 g/cm2, p < 0.0001) and total body (0.96 vs. 1.02 g/cm2, *p* < 0.0001); meanwhile, there was no difference in BMD for lumbar spine. (ii) There was no evident difference between former smokers and never-smokers in any of the skeletal regions assessed by DXA or ultrasound.

Oncken et al, 2002 [88]	Randomized-placebo-controlled study, subjects followed for 6 weeks	(i) N = 66, after 6 weeks of follow up the analysis includes: 20 quitter from smoking cessation group, and 18 from wait-list control group (ii) All participants were females	(i) To assess effects smoking cessation on hormone profiles and bone turnover markers in postmenopausal women.	(i) After 6 weeks, compared with wait-list control group, smoking cessation group had a significant change in N-terminal collagen cross-links (NTx) (-5% vs. +56%, respectively, *p* = 0.01) and sex hormone binding globulin (SHBG) (-8% vs. +5%, respectively; *p* = 0.01). (ii) There was significant correlation between plasma cotinine and SHBG (r = 0.48; *p *= 0.004) and NTx (r = 0.36; *p* = 0.04).

Oncken et al, 2006 [89]	Randomized-placebo-controlled study, subjects followed for 1 year	(i) N = 152 (smoked 10 or more cigarettes/day at baseline), after 1 year of follow-up the analysis includes 42 quitter, and 77 continued smoking (ii) All participants were females	(i) To examine effects of smoking cessation on BMD, bone turnover markers, and hormone profiles in postmenopausal women.	(i) The BMD of femoral trochanter was significantly increased among quitter than that who continued to smoke (2.9% vs. 0.6%, *p* = 0.02). Same finding was seen for BMD of total hip (1.52% vs. 0.43%, *p* = 0.03). However there were no significant changes observed at femoral neck, radius, spine, and total body. (ii) Smoking cessation was associated with an increase in bone alkaline phosphatase.
